# Antiplasmodial Alkaloids from the Bark of *Cryptocarya nigra* (Lauraceae)

**DOI:** 10.3390/molecules18078009

**Published:** 2013-07-08

**Authors:** Ayu Afiqah Nasrullah, Azeana Zahari, Jamaludin Mohamad, Khalijah Awang

**Affiliations:** 1 Department of Chemistry, Faculty of Science, University of Malaya, 50603 Kuala Lumpur, Malaysia; E-Mails: ayuafiqah13@siswa.um.edu.my (A.A.N.); ezianna@gmail.com (A.Z.); 2 Institute of Biological Sciences, Faculty of Science, University of Malaya, 50603 Kuala Lumpur, Malaysia; E-Mail: jamal@um.edu.my

**Keywords:** *Plasmodium falciparum*, *Cryptocarya nigra*, Lauraceae, antioxidant, antiplasmodial, activity

## Abstract

A dichloromethane extract of the stem bark of *Cryptocarya nigra* showed strong *in vitro* inhibition of *Plasmodium falciparum* growth, with an IC_50_ value of 2.82 μg/mL. The phytochemical study of this extract has led to the isolation and characterization of four known alkaloids: (+)-*N*-methylisococlaurine (**1**), atherosperminine (**2**), 2-hydroxyathersperminine (**3**), and noratherosperminine (**4**). Structural elucidation of all alkaloids was accomplished by means of high field 1D- and 2D-NMR, IR, UV and LCMS spectral data. The isolated extract constituents (+)-*N*-methylisococlaurine (**1)**, atherosperminine (**2**) and 2-hydroxy-atherosperminine (**3**) showed strong antiplasmodial activity, with IC_50_ values of 5.40, 5.80 and 0.75 μM, respectively. In addition, (+)-*N*-methylisocolaurine (**1**) and atherosperminine (**2**) showed high antioxidant activity in a DPPH assay with IC_50_ values of 29.56 ug/mL and 54.53 ug/mL respectively. Compounds **1** and **2** also both showed high antioxidant activity in the FRAP assay, with percentages of 78.54 and 70.66 respectively and in the metal chelating assay, with IC_50_ values of 50.08 ug/mL and 42.87 ug/mL, respectively.

## 1. Introduction

Malaria is caused by five species of parasites from the genus *Plasmodium*: *P. falciparum*, *P. vivax*, *P. ovale*, *P. malariae*, *P. knowlesi* and *P. falciparum* is the most deadly form that affects humans [1]. According to the latest data from the WHO, the estimated incidence of malaria has remained unacceptably high. There were about 219 million cases of malaria in 2010 and an estimated 660,000 deaths [2]. A major factor in the continuing burden of malaria is the spread of parasites resistant to front-line anti-malarials such as chloroquine and sulphadoxine/ pyrimethamine, and artemisinin [3].

Malaysia, being host of one of the oldest and richest forest ecosystems in the World with a reported more than 15,000 plant species is an excellent source of natural bioactive compounds [4]. In our continuing efforts to search for new antiplasmodial agents [5,6,7,8,9,10], we report herein our phytochemical studies on *Cryptocarya nigra* (Lauraceae), whose dichloromethane extract showed potent antiplasmodial activity with an IC_50_ value 2.82 μg/mL.

This plant is widely distributed in Peninsular Malaysia, Sumatra and Borneo, and it is locally known as ‘*Medang*’ [11]. The genus *Cryptocarya* belonged to the family Lauraceae which comprises of more than 350 species of which 19 species are found in Malaysia [12,13]. This species is a medium sized tree that grows up to 10 m tall. Based on previously reported phytochemical and pharmacological studies, the genus *Cryptocarya* are known to be prolific producers of flavonoids, chalcones, lactones, α-pyrones and mainly alkaloids [14,15,16,17,18,19,20,21,22,23,24] with varied biological activities. A preliminary study on the leaves of the *Cryptocarya nigra* reported the presence of coumarins and flavonoids and their cytotoxic activity against murine leukemia P-388 cells [25]. However, no study has been reported on the alkaloidal content, antiplasmodial and antioxidant activities of *Cryptocarya nigra* extracts

**Figure 1:**
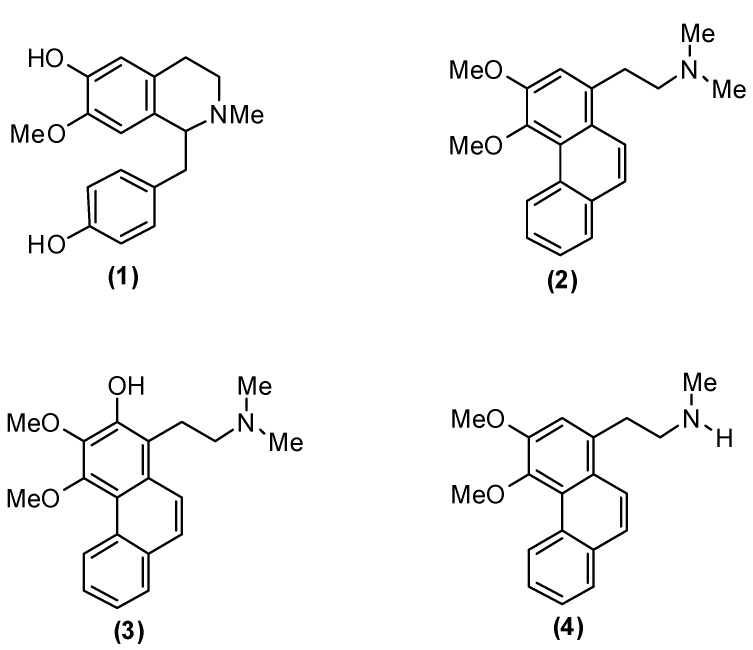
Structures of compounds **1**–**4**.

The phytochemical study on the stem bark of *Cryptocarya nigra* has now led to the isolation of four known alkaloids; (+)-*N*-methylisococlaurine (**1**) [26], atherosperminine (**2**) [27], 2-hydroxyathersperminine (**3**) [24], and noratherosperminine (**4**) [28] (Figure 1). Alkaloids **1**–**3** were evaluated for their antiplasmodial activity and alkaloids **1** and **2** were also subjected to antioxidant assays.

## 2. Results and Discussion

Extensive chromatographic analysis of the dicholoromethane extract (DCE) of the bark of *Cryptocarya nigra* afforded one benzylisoquinoline alkaloid, (+)-*N*-methylisococlaurine (**1**), and three phenanthrene alkaloids: atherosperminine (**2**), 2-hydroxyathersperminine (**3**), and noratherosperminine (**4**). The structure of each compound was characterized by analysis of its spectroscopic data and by comparison of its NMR, IR, UV and MS data with literature values [24,26,27,28].

Phenanthrene alkaloids are derivatives of phenanthrene with a 1-(2-aminoethyl) side chain. They are a very rare type also known as “seco-aporphine” alkaloids, probably formed biogenetically from an aporphine precursor through opening of ring B [29]. Phenanthrene alkaloids have been reported to exhibit antimicrobial activity, cytotoxicity, and dopamine receptor stimulation effects [27,30,31].

### 2.1. Antiplasmodial Activity

The compounds isolated from the bark of *Cryptocarya nigra* were tested for antiplasmodial activity based on the promising screening results of the DCE (IC_50_ = 2.82 μg/mL). The *in vitro* antiplasmodial activity of compounds isolated from *Cryptocarya nigra* against a chloroquine resistant strain of *P. falciparum* (K1 strain) is summarized in Table 1. Compound **3** displayed the strongest inhibition activity, with an IC_50_ value of 0.75 μM, followed by compound **1** and compound **2** with IC_50_ values of 5.40 μM and 5.80 μM, respectively. These alkaloids have no previous record of any antiplasmodial activity. 

**Table 1:** *In vitro* Antiplasmodial Activities of Alkaloids from *Cryptocarya nigra*.

Sample	Antiplasmodial Activity * The % of growth inhibition or IC_50_	
	μg/mL	μM
**1**	1.62	5.40
**2**	1.80	5.80
**3**	0.25	0.75
**4**	nt ^†^	nt ^†^
Chloroquine	90.39 ± 28.85 **	
Artemisinin	2.42 ± 1.06 **	

* Results are recorded as the % of growth inhibition or IC_50_. Notes: nt ^†^—not tested; ** unit in nM.

### 2.2. Antioxidant Activity

Compound **1**, a benzylisoquinoline, showed high scavenging activity towards the free radical DPPH at IC_50_ = 29.56 µg/mL and high reducing power at 78.54% as compared to compound **2** (Table 2). However compound **2** showed higher metal chelating activity than compound **1** at IC_50_ = 42.87 ug/mL and IC_50_ 50.08 ug/mL, respectively. The high DPPH and FRAP antioxidant activity of compound **1** may be due to its hydroxyl group that could donate an electron to free radicals and possesses the ability to chelate metals. In addition, Jee reported that the phenanthrenes have shown an effect on glutathione reductase that increases antioxidant activity in the fish *Paralichthys olivaceus* (olive flounder) [32]. Thus, compound **1** and compound **2** are good reductants with the ability to chelate metals and presented pro-oxidant activity.

**Table 2:** Antioxidant Activities of Alkaloids from *Cryptocarya nigra*.

Compound Name	IC_50_ DPPH Activity (ug/mL)	% FRAP	IC_50_ Metal Chelating Activity (ug/mL)
*N*-Methylisococlaurine (**1**)	29.56	78.54	50.08
Atherosperminine (**2**)	54.53	70.66	42.87
Ascorbic acid (Standard)	13.69		
EDTA (Standard)		83.74	
BHA (Standard)			19.60

The results showed that compounds **1** and **2** exhibited both iron binding and antiplasmodial activity. This suggests that there is iron requirement in host-parasite interactions [33,34] for oxygen transport, respiration and enzymes activities. These results indicate that there may be a correlation between antiplamodial activities and iron chelating in metal chelating [35]. Thus, metal chelating ability has potential in antimalarial treatment. The mechanism(s) of action of iron chelators are still not known and further investigation must be carried out on compounds **1** and **2** in order to elucidate their metal chelating mechanism and their potential as antimalarial agents. The antioxidant effect might play a major role in inhibiting the actions of the parasites, but it may also stimulate the immune system of malarial victims [36], therefore being a good candidate for antimalarial therapy.

According to Reis *et al.*, the combination of chloroquine and two antioxidant agents, prevented both inflammatory and vascular changes in the tissues of the brain at the first signs of cerebral malaria, as well as the development of persistent cognitive damage. The addition of antioxidants did not diminish the efficacy of chloroquine in eliminating *Plasmodia* from the blood. The authors suggested that these antioxidant drugs should be studied as an additive therapy for antimalarial drugs in clinical trials in order to investigate their potential to reduce or prevent cognitive damage after cerebral malaria [37]. Additionally, recent studies by Percario *et al.* on oxidative stress in malaria [38] have suggested that the use of antioxidant supplements of synthetic or natural origin may constitute a far more effective adjuvant antimalarial strategy that causes less damage to the host.

## 3. Experimental

### 3.1. General

Spectra were recorded on the following instruments: UV: Shimadzu UV-250 UV-Visible spectrophotometer; IR: Perkin Elmer 1600; NMR: JEOL ECA 400 MHz; LCMS-IT-TOF: Shimadzu. All solvents, except those used for bulk extraction were AR grade. Silica gel 60 F254 was used for column chromatography. Glass and aluminum-supported silica gel 60 F254 plates were used for preparative TLC. TLC spots were visualized under UV light (254 and 365 nm) followed by spraying with Dragendorff’s reagent for alkaloid detection.

### 3.2. Plant Material

The bark of *Cryptocarya nigra* was collected at Hutan Simpan Ulu Sat, Machang, Kelantan (Malaysia) by the phytochemical group of the Department of Chemistry, Faculty of Science, University of Malaya. A voucher specimen (KL5272) has been deposited at the Herbarium of the Department of Chemistry, University Malaya, Kuala Lumpur, Malaysia.

### 3.3. Extraction and Isolation

The air-dried ground bark of the plant (2.0 kg) was first exhaustively extracted twice with hexane (14 L) for a 3-day period. The hexane extracts were combined and the solvent evaporated. The dried plant material were then made alkaline and moistened with 25% NH_4_OH (1 L) for 2 h. It was then macerated with CH_2_Cl_2_ (14 L) twice for a 3-day period. After filtration, the supernatant obtained was concentrated under reduced pressure using a rotary evaporator to a volume of 500 mL and tested for alkaloid content (using TLC and confirmed by spraying with Dragendorff’s reagent). The extract was finally concentrated and dried to give 15.0 g of extract designated as the DCE. The crude alkaloid extract (8.0 g) was subjected to column chromatography over silica gel using CH_2_Cl_2_ and methanol mixtures (100:0, 99:1, 98:2, 97:3, 96:4, 95:5, 90:10, 80:20, and 70:30) and finally 100% methanol as eluent to obtain twenty fractions. Further purification of fraction 16 by Preparative Thin Layer Chromatography (PTLC) yielded major alkaloid **1** (25.0 mg, MeOH-CH_2_Cl_2_; 95:5: saturated with NH_4_OH). In addition, known compounds **2** (15.0 mg, MeOH-CH_2_Cl_2_; 96:4: saturated with NH_4_OH), **3** (5.0 mg, MeOH-CH_2_Cl_2_; 96:4: saturated with NH_4_OH), and **4** (1.5 mg, MeOH-CH_2_Cl_2_; 98:2: saturated with NH_4_OH) were obtained after purification of fraction 17.

### 3.4. Determination of Antiplasmodial Activity

The samples were sent to the Institute for Medical Research, Kuala Lumpur (IMR) for antiplasmodial screening. DCE and the isolated compounds were assayed for *in vitro* antiplasmodial activity against the *Plasmodium falciparum*, K1 isolate (resistant strain). Briefly the methodology involves malaria culture as discussed by Trager and Jensen [39] with some modifications. The synchronization of the malaria culture to one stage is according to Lambros and Vandernerg [40]. Chloroquine and artemisinin were used as positive controls. The antiplasmodial activity of each compound was expressed as an IC_50_ value, defined as the concentration of the compound causing 50% inhibition of parasite growth relative to an untreated control.

### 3.5. Determination of Antioxidant Assay

#### 3.5.1. DPPH Assay

The DPPH antioxidant assay was determined as described by Shimada *et al.* [41]. Briefly, 0.1 mM DPPH (1 mL) dissolved in ethanol was added to an ethanol solution (3 mL) of the tested compound at different concentrations (0, 50, 100, 150, 200 µg/mL). An equal volume of ethanol was added in the control test. The mixture was shaken vigorously and allowed to stand at room temperature for 30 min. Then the absorbance at 517 nm was measured with a UV–VIS spectrophotometer. The percentage of scavenging of DPPH was calculated using the following equation:

(1) $$\text{DPPH scavenging effect }(\%)=\frac{A^{{}^{\circ}}-A1}{A^{{}^{\circ}}}\times 100$$

where A^o^ is the absorbance of the control reaction and A1 is the absorbance in the presence of the sample.

#### 3.5.2. Ferric Reducing Power Assay (FRAP)

The reducing power was determined using the method of Oyaizu [42]. The tested compounds (0.5 mL) dissolved in ethanol at different doses (0, 50, 100, 150, 200 µg/mL) were mixed with phosphate buffer (0.5 mL, 0.2 M, pH 6.6) and potassium ferricyanide [K3Fe(CN)6] (0.5 mL, 1%). The mixture was then incubated at 50 °C for 20 min. A portion of trichloroacetic acid (0.5 mL, 10%) was added to the mixture, which was then centrifuged for 10 min at 3000 rpm (1000 g). The upper layer of solution (0.5 mL) was mixed with distilled water (0.5 mL) and FeCl_3_ (0.1 mL, 0.1%) for 10 min, and then the absorbance was measured at 700 nm in a spectrophotometer. EDTA at different concentrations (2.5 µg/mL to 40 μg/mL) was used as standard reference. The reducing power activity was expressed as percentage of absorbance compared with EDTA.

#### 3.5.3. Metal Chelating Activity Assay

The chelation of ferrous ions was estimated according to the method of Dinis *et al.* [43]. Briefly, tested compounds dissolved in ethanol (0.95 mL, at different doses of 0, 50, 100, 150, 200 µg/mL) were added to a solution of FeCl_2_ (2 mM, 50 µL). The reaction was initiated by the addition of ferrozine (5 mM, 200 µL), and then the mixture was shaken vigorously and center standing at room temperature for 10 min. After equilibrium had been reached, absorbance of the solution was measured spectrophotometrically at 562 nm. The percentage of inhibition of ferrozine-Fe^2+^ complex of each sample was calculated according to the following formula:

% inhibition = [(Abs_control_ – Abs_sample_)/Abs_control_] × 100 (2)

where, Abs_control_ = absorbance reading of control and Abs_sample_ = absorbance reading of sample. The IC_50_ value was determined from the graph of percentage inhibition against concentration.

## 4. Conclusion

A phytochemical study on dried bark of *Cryptocarya nigra* afforded four known alkaloids **1**–**4**. The alkaloids (+)-*N*-methylisococlaurine (**1**), atherosperminine (**2**) and 2-hydroxyatherosperminine (**3**) strongly inhibited *in vitro* growth of a chloroquine resistant strain of *Plasmodium falciparum* (K1 strain), with the strongest inhibition shown by compound **3**. In addition, both compounds **1** and **2** exhibited good antioxidant activity, therefore these compounds could be potential candidates as antimalarial agents since antioxidants are said to be an additive therapy for malaria. The antiplasmodial and antioxidant data suggested that the bark extract of *Cryptocarya nigra* is a potential source of new antiplasmodial agents having antioxidant potential.
